# A force sensor improves trainee technique for rigid endoscopy

**DOI:** 10.1017/S0022215124000057

**Published:** 2024-06

**Authors:** Sumrit Bola, Manu Kunaal Shrivastava, Josh Brown, Matthew Cherko, Beatrice Emmanouil

**Affiliations:** 1 ENT Department, Oxford University Hospitals NHS Foundation Trust, Oxford, UK; 2 Department of Psychology, Health and Professional Development, Faculty of Health and Life Sciences, Oxford Brookes University, Oxford, UK

**Keywords:** Endoscopy, head and neck cancer, dysphagia

## Abstract

**Objective:**

Developing skills in rigid endoscopy poses challenges to the surgical trainee. This study investigates whether a modified manikin can improve the technical skill of junior operators by providing direct quantitative feedback.

**Methods:**

A force-sensing pad was incorporated into the oral cavity of a life support manikin. Junior trainees and senior otolaryngologists were invited to perform rigid endoscopy and received real-time feedback from the force sensor during the procedure.

**Results:**

There was a significant inverse correlation between operator seniority and the weight applied to the oral cavity (*p* < 0.0001). All junior trainee operators applied less weight after five attempts (346 ± 90.95 g) compared to their first attempt (464 ± 85.79 g). This gave a statistically significant decrease of 118 g (standard deviation = 107.27 g, *p* = 0.007) when quantitative feedback was provided to learning operators.

**Conclusion:**

This low-cost, simple model allows trainees to rehearse a high-risk procedure in a safe environment and adjust their operative technique.

## Introduction

Rigid endoscopy is a widely used diagnostic and therapeutic procedure. Major complications, such as oesophageal perforation and gastrointestinal bleeding, are well documented in the literature and can lead to significant morbidity.^1^ Injury to the oral cavity occurs in up to 37 per cent of patients,^2^ and resultant damage to the teeth and oral mucosa can be costly and cause a delayed return to normal diet.

Developing skills in rigid endoscopy poses challenges to the surgical trainee. There is a restricted view for the trainer, and it is difficult to ascertain how well the trainee is avoiding injury to the oral cavity, dentition and oesophagus. This compromises the trainer's ability to provide constructive intra-operative feedback. These challenges are on a background of reduced operative numbers within surgical training across all specialties; this is something that has been addressed by a greater emphasis on competency-based assessments and simulation, to enable trainee progression and provide evidence of surgical competence.^3–6^

Manikin simulators offer a unique training advantage in creating an immersive learning experience. Procedural skills can be repeated and standardised in a low-stress environment, without risking patient safety. Over the past two decades, manikin simulators have become more sophisticated, and can now mimic physiological states, facilitate life support training and provide higher fidelity simulation.^7,8^ They also assist in the development of psychomotor skills and have been used in a variety of different surgical specialties.^9–14^ Interestingly, although procedures that risk the oral cavity (intubation laryngoscopy, bronchoscopy and rigid endoscopy) are common, no mainstream oral sensors have been developed. Some manikins allow for auditory feedback with ‘clicks’ if force is applied to the teeth, but this does not help trainees moderate force whilst performing the procedure. A literature review showed that one group had used force sensors in a plaster and silicon-based model of the mouth and oesophagus; it was demonstrated that the pressure exerted by trainees during rigid endoscopy was inversely correlated with the level of experience.^15^

We test a training model where rigid endoscopy is performed on a low-fidelity (basic) manikin after the insertion of an oral force sensor. The aims of the training were to: (1) improve awareness of force application whilst using the scope; and (2) investigate if real-time force feedback could result in adaptation of technique to lessen the force applied to the oral cavity.

## Materials and methods

**Figure 1.**
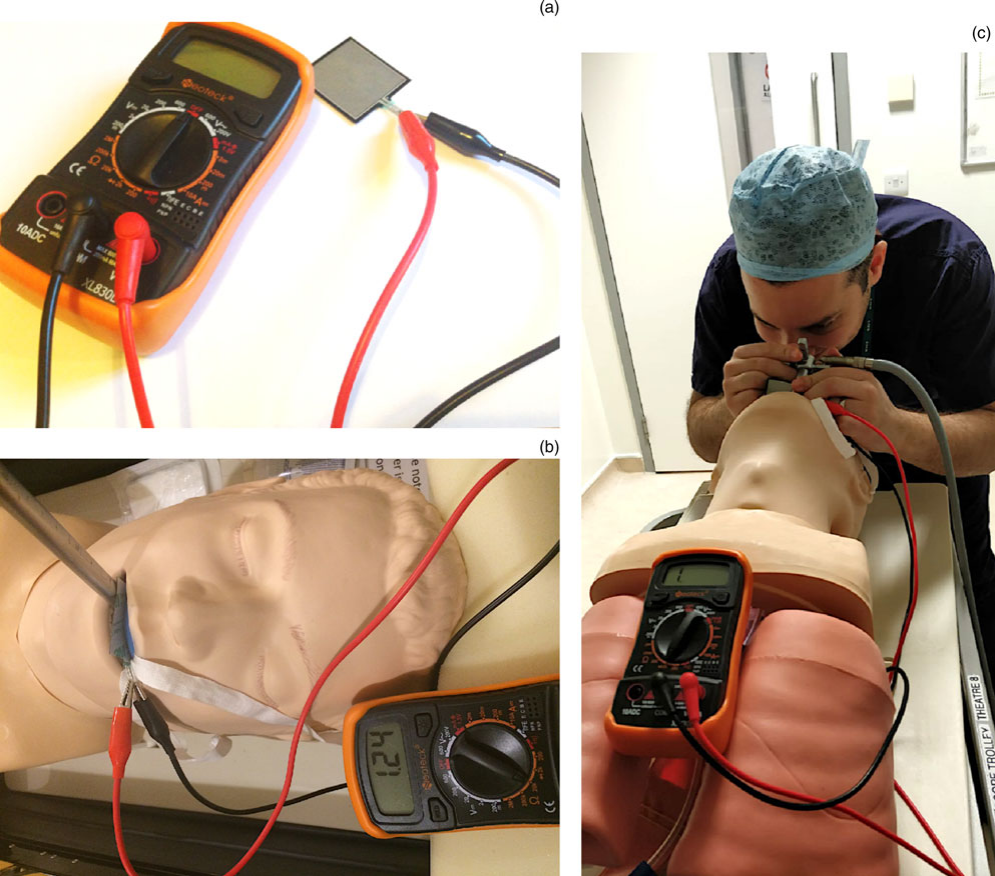
(a) Force sensitive resistor connected to a multimeter. (b) Sensor placed on gum guard of the manikin. (c) Trainee performing simulated rigid oesophagoscopy on a manikin.

A high accuracy, thin-film, force sensitive resistor (model RP-S40-ST; costing £11.50, capable of detecting weights of 20–10 000 g) was connected to a multimeter (Neoteck Pocket Multimeter; costing £14.99) (Figure 1). The force sensitive resistor pad increases its resistance (in ohms) when force is applied. This is measured by the multimeter, which was set to a baseline resistance of 2 kΩ to allow for measurable recordings. As the relationship between resistance and force is logarithmic, different weights were tested on the sensor, and measurements of the resistance were used to form a simple calibration curve. In order to provide a relatable metric for trainees, we provided feedback in weight (grams) from the calibration curve; however, different metrics can be used if the model is replicated. The study protocol was granted ethical approval at the department's clinical governance meeting and no ethical conflicts were identified. All procedures contributing to this work complied with local clinical governance guidelines.

The force sensitive resistor sensing pad was attached to a gum guard inserted on the upper teeth of a manikin, with part of it resting on the palate (Figure 1). Rigid endoscopy was performed using a standard 30 cm adult oesophagoscope and light lead. The operator was able to visualise the reading from the multimeter as the oesophagoscope moved along the upper teeth.

Endoscopy was performed on the manikin by consultants, registrars and junior trainees. The juniors had never performed the procedure and had graduated from medical school within the previous three years. Without coaching, the junior trainees were given five attempts at intubating the oesophagus. The ‘live’ readings from the force sensitive resistor and multimeter enabled them to see whether the weight they exerted on the upper teeth could be improved, with direct numerical feedback (Figure 1). They were told the readings of the senior participants.

A paired samples *t*-test was used to determine if there was a statistically significant improvement in the weight applied to the manikin's oral cavity by trainees after five attempts. Data are expressed as mean ± standard deviation, unless otherwise stated. The assumption of normality was not violated, as assessed by the Shapiro–Wilk test (*p* = 0.135). Spearman's rank-order correlation was used to assess the relationship between doctors’ seniority and weight recorded at the first attempt.

## Results

**Figure 2.**
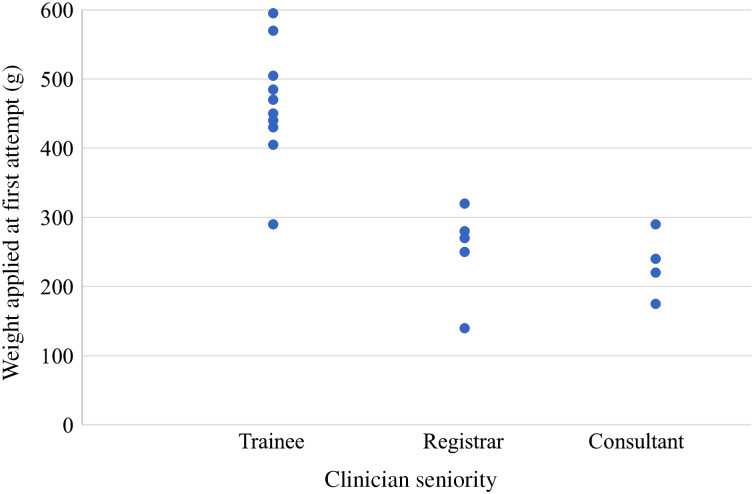
Scatterplot of results demonstrating the weight recorded at the first attempt at simulated rigid oesophagoscopy for trainees, registrars and consultants. Each dot represents a participant.

The training exercise was performed by 19 operators, divided into three categories: 10 junior trainees (foundation year trainees or core surgical trainees), 5 registrars (specialist otolaryngology trainees) and 4 consultants (head and neck specialists). There was a statistically significant, strong negative correlation between operator seniority and the weight applied to the oral cavity on the first attempt (rs (17) = −0.824, *p* < 0.0001). Visual inspection of a scatterplot (Figure 2) showed the relationship to be monotonic; the more experienced clinicians applied less weight on the manikin's oral cavity.

**Figure 3.**
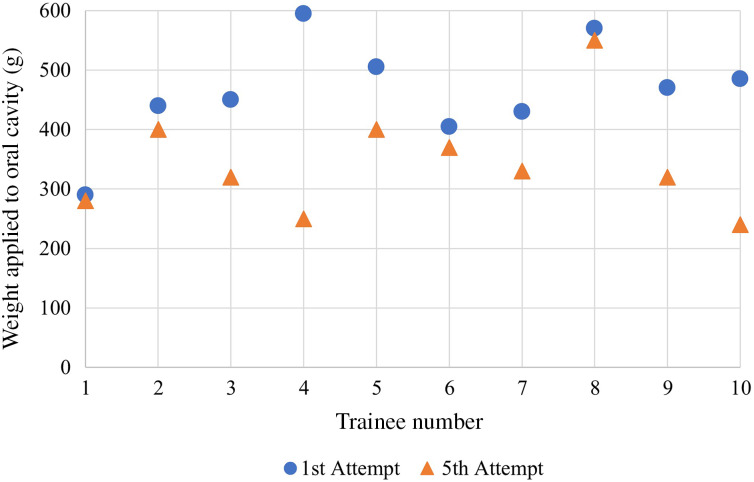
Scatterplot demonstrating the weight recorded by each of the 10 junior trainees at their first and fifth attempts.

All junior trainee operators applied less weight after five attempts (Figure 3) (346 ± 90.95 g) compared to their first attempt (464 ± 85.79 g). This gave a statistically significant decrease of 118 g (standard deviation = 107.27) after five attempts (*t* (9) = 3.479, *p* = 0.007, d = 1.1).

The operators' technique was observed during the simulation, and without external coaching or verbal feedback. The junior operators made observable adaptations to their technique with repeated attempts. Observed behaviours included: extending the neck of the manikin; using the thumb of their non-dominant hand as a fulcrum for the scope; following the lateral corner of the mouth and lateral wall of the hypopharynx; and readjusting to the midline and moving to a sitting position.

## Discussion

Damage to the oral cavity during rigid endoscopy can lead to expensive dental work, bleeding and difficulty wearing dentures post-operatively. Moreover, it is likely that applying more force (or weight) at the proximal end of the scope is related to increased force distally, risking injury to the delicate oesophageal mucosa.

This training model was easy to set up, and it familiarised the junior trainee with hypopharyngeal anatomy and the intended straight axis between the mouth, pharynx and oesophagus. The trainee needed to adjust their technique to exert less force on the oral cavity, and this increased the fidelity of the training manikin. Whilst previous work has shown that the pressure exerted in rigid oesophagoscopy is inversely correlated with experience,^15^ this study is the first to analyse the educational potential of real-time feedback in a pressure sensor model. The model uses manikins, which are available in most hospitals, making this a simple training exercise to recreate. We witnessed a statistically significant reduction in the weight applied to the manikin's upper teeth and oral cavity after five attempts. The improved readings became closer to the readings of the senior technician groups (registrars and consultants). It is possible that eliminating trainer feedback whilst in a safe environment reduces cognitive load, so that the trainee can better access theoretical knowledge.

This study was limited by only having a sensor in the upper oral cavity, and in providing a manikin with a ‘standard’ oesophagus, which may not reflect the strictures, pouches, osteophytes and other anatomical variations that can be found in real life. In addition, although force sensitive resistors are good for obtaining a rough measurement of force or weight, trainees must create a calibration curve by plotting different weights against the multimeter reading (e.g. 0 kg on force sensitive resistor = reading of 1) if they want a relatable reference, although this is not necessary for plotting improvements.

## Conclusion

Simulation without external coaching creates a low-stress environment, allowing the trainee to access theoretical knowledge. Low-cost force-sensing equipment can enhance the fidelity of manikins in rigid endoscopy. Real-time quantitative feedback can be a sufficient starting point in teaching trainees to adjust their operative technique, and may be considered in other procedures such as bronchoscopy or laryngoscopy.
